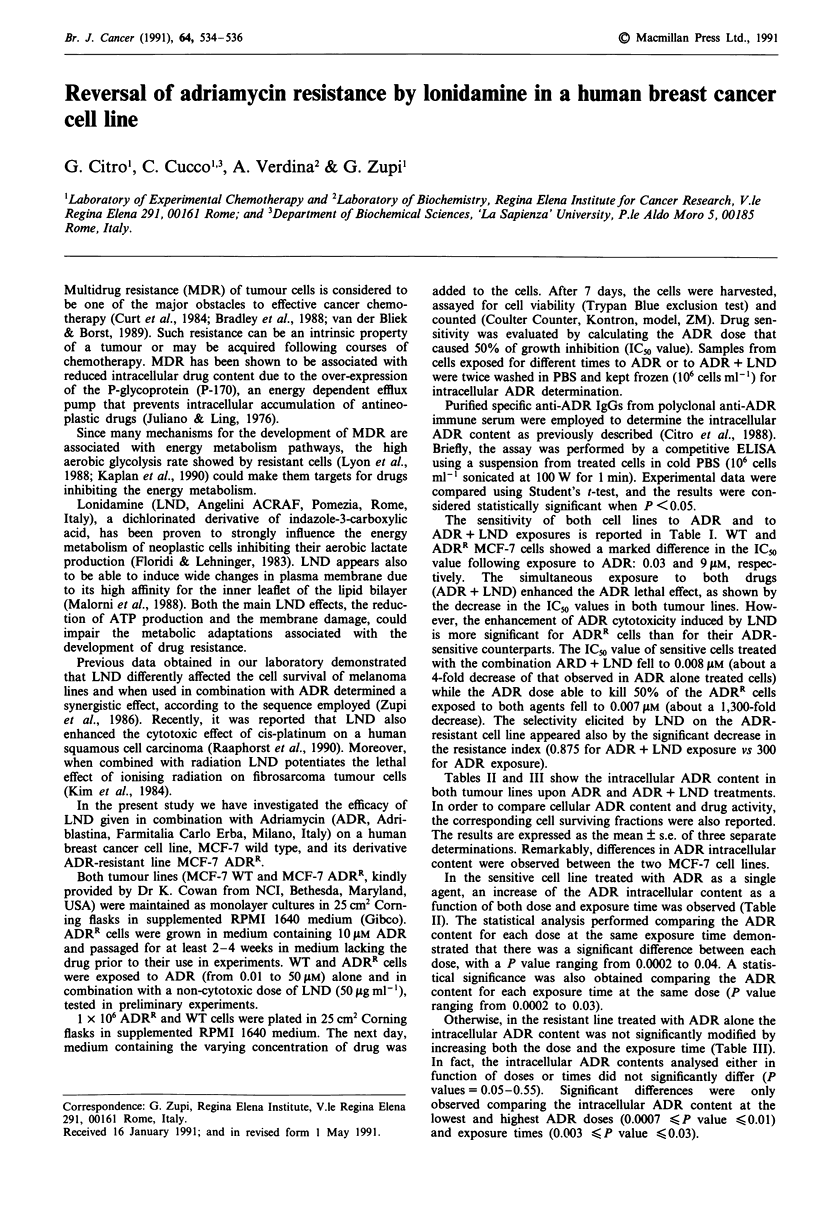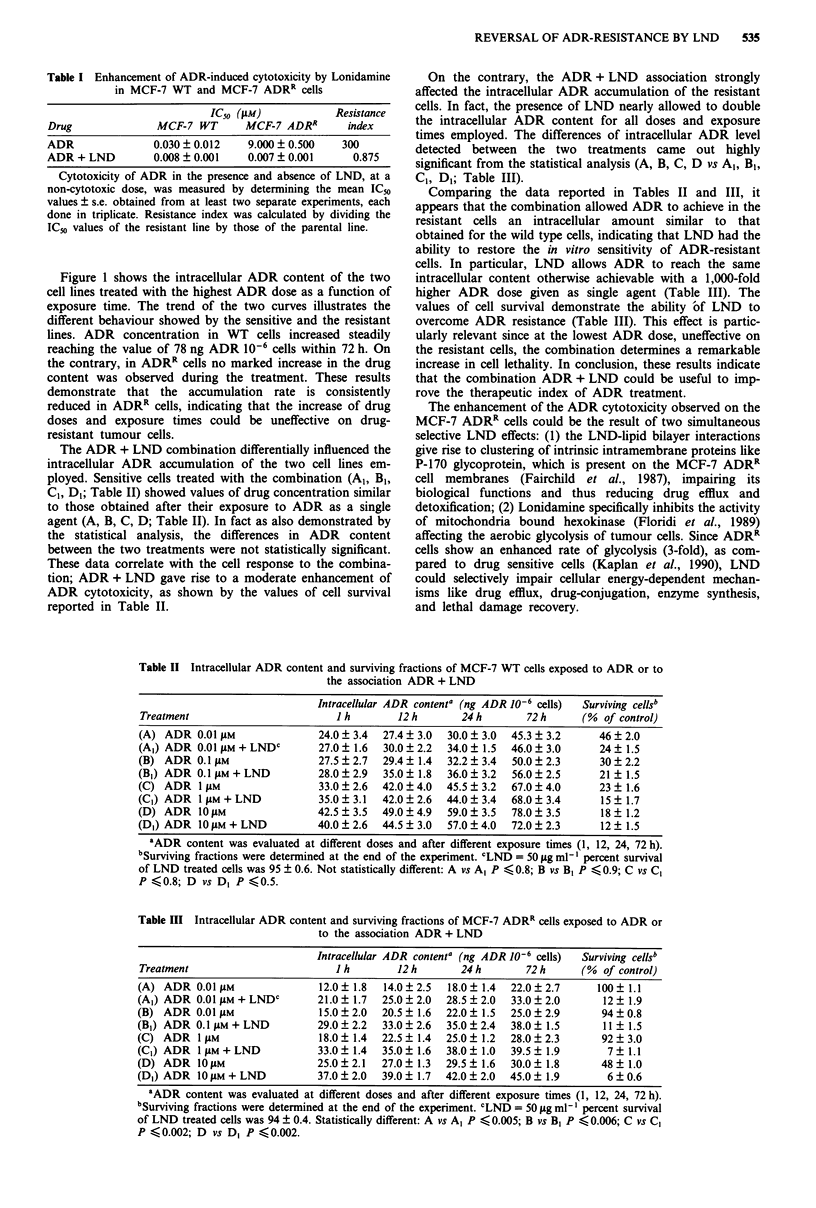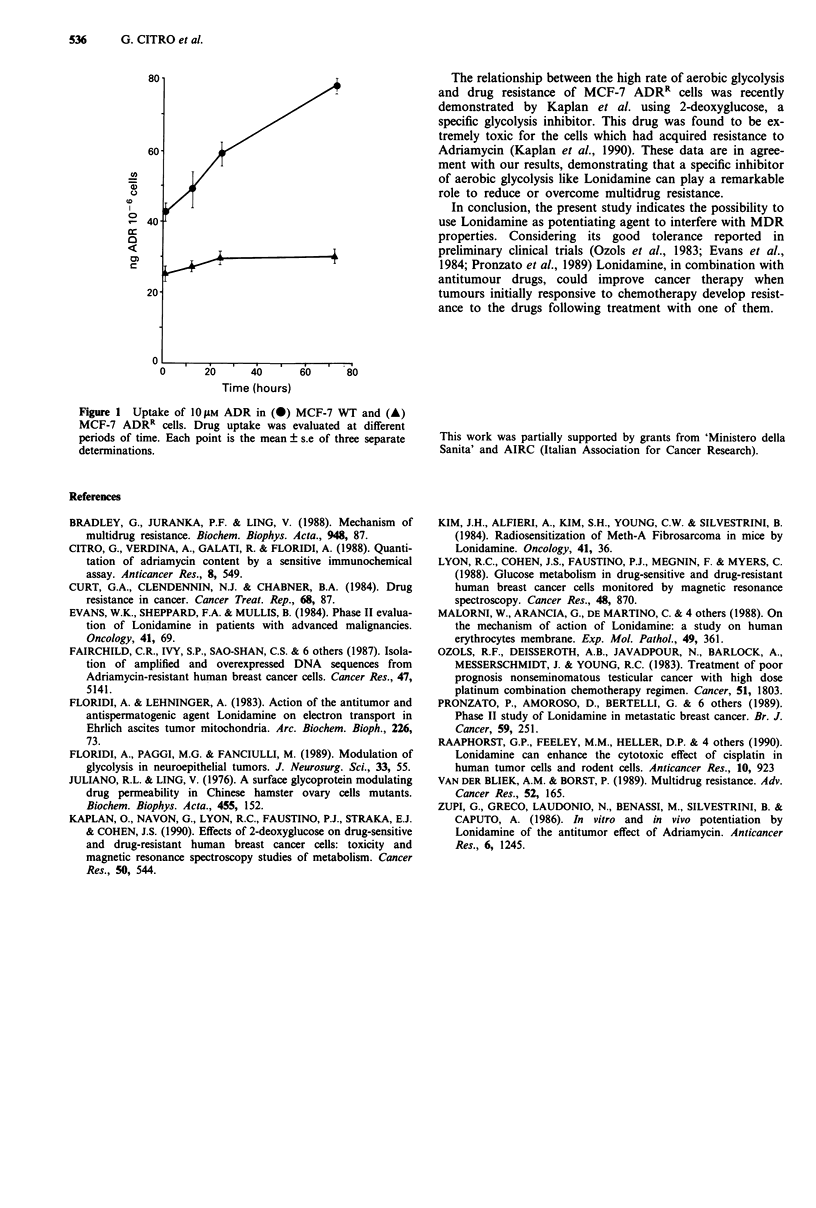# Reversal of adriamycin resistance by lonidamine in a human breast cancer cell line.

**DOI:** 10.1038/bjc.1991.345

**Published:** 1991-09

**Authors:** G. Citro, C. Cucco, A. Verdina, G. Zupi

**Affiliations:** Laboratory of Experimental Chemotherapy, Regina Elena Institute for Cancer Research, Rome, Italy.


Br. J. Cancer (1991), 64, 534-536 © Macmillan Press Ltd., 1991

### Reversal of adriamycin resistance by lonidamine in a human breast cancer cell line

G. Citro[1], C. Cucco[1,3], A. Verdina[2] & G. Zupi[1]

[1]*Laboratory of Experimental Chemotherapy and* [2]*Laboratory of Biochemistry, Regina Elena Institute for Cancer Research, V.le Regina Elena 291, 00161 Rome; and* [3]*Department of Biochemical Sciences, 'La Sapienza' University, P.le Aldo Moro 5, 00185 Rome, Italy.*

Multidrug resistance (MDR) of tumour cells is considered to be one of the major obstacles to effective cancer chemotherapy (Curt *et al.*, 1984; Bradley *et al.*, 1988; van der Bliek & Borst, 1989). Such resistance can be an intrinsic property of a tumour or may be acquired following courses of chemotherapy. MDR has been shown to be associated with reduced intracellular drug content due to the over-expression of the P-glycoprotein (P-170), an energy dependent efflux pump that prevents intracellular accumulation of antineoplastic drugs (Juliano & Ling, 1976).

Since many mechanisms for the development of MDR are associated with energy metabolism pathways, the high aerobic glycolysis rate showed by resistant cells (Lyon *et al.*, 1988; Kaplan *et al.*, 1990) could make them targets for drugs inhibiting the energy metabolism.

Lonidamine (LND, Angelini ACRAF, Pomezia, Rome, Italy), a dichlorinated derivative of indazole-3-carboxylic acid, has been proven to strongly influence the energy metabolism of neoplastic cells inhibiting their aerobic lactate production (Floridi & Lehninger, 1983). LND appears also to be able to induce wide changes in plasma membrane due to its high affinity for the inner leaflet of the lipid bilayer (Malorni *et al.*, 1988). Both the main LND effects, the reduction of ATP production and the membrane damage, could impair the metabolic adaptations associated with the development of drug resistance.

Previous data obtained in our laboratory demonstrated that LND differently affected the cell survival of melanoma lines and when used in combination with ADR determined a synergistic effect, according to the sequence employed (Zupi *et al.*, 1986). Recently, it was reported that LND also enhanced the cytotoxic effect of cis-platinum on a human squamous cell carcinoma (Raaphorst *et al.*, 1990). Moreover, when combined with radiation LND potentiates the lethal effect of ionising radiation on fibrosarcoma tumour cells (Kim *et al.*, 1984).

In the present study we have investigated the efficacy of LND given in combination with Adriamycin (ADR, Adriblastina, Farmitalia Carlo Erba, Milano, Italy) on a human breast cancer cell line, MCF-7 wild type, and its derivative ADR-resistant line MCF-7 $ADR^R$.

Both tumour lines (MCF-7 WT and MCF-7 $ADR^R$, kindly provided by Dr K. Cowan from NCI, Bethesda, Maryland, USA) were maintained as monolayer cultures in 25 $cm^2$ Corning flasks in supplemented RPMI 1640 medium (Gibco). $ADR^R$ cells were grown in medium containing 10 $\mu$M ADR and passaged for at least 2–4 weeks in medium lacking the drug prior to their use in experiments. WT and $ADR^R$ cells were exposed to ADR (from 0.01 to 50 $\mu$M) alone and in combination with a non-cytotoxic dose of LND (50 $\mu g\ ml^{-1}$), tested in preliminary experiments.

$1 \times 10^6$ $ADR^R$ and WT cells were plated in 25 $cm^2$ Corning flasks in supplemented RPMI 1640 medium. The next day, medium containing the varying concentration of drug was added to the cells. After 7 days, the cells were harvested, assayed for cell viability (Trypan Blue exclusion test) and counted (Coulter Counter, Kontron, model, ZM). Drug sensitivity was evaluated by calculating the ADR dose that caused 50% of growth inhibition ($IC_{50}$ value). Samples from cells exposed for different times to ADR or to ADR + LND were twice washed in PBS and kept frozen ($10^6$ cells $ml^{-1}$) for intracellular ADR determination.

Purified specific anti-ADR IgGs from polyclonal anti-ADR immune serum were employed to determine the intracellular ADR content as previously described (Citro *et al.*, 1988). Briefly, the assay was performed by a competitive ELISA using a suspension from treated cells in cold PBS ($10^6$ cells $ml^{-1}$ sonicated at 100 W for 1 min). Experimental data were compared using Student's *t*-test, and the results were considered statistically significant when $P < 0.05$.

The sensitivity of both cell lines to ADR and to ADR + LND exposures is reported in Table I. WT and $ADR^R$ MCF-7 cells showed a marked difference in the $IC_{50}$ value following exposure to ADR: 0.03 and 9 $\mu$M, respectively. The simultaneous exposure to both drugs (ADR + LND) enhanced the ADR lethal effect, as shown by the decrease in the $IC_{50}$ values in both tumour lines. However, the enhancement of ADR cytotoxicity induced by LND is more significant for $ADR^R$ cells than for their ADR-sensitive counterparts. The $IC_{50}$ value of sensitive cells treated with the combination ARD + LND fell to 0.008 $\mu$M (about a 4-fold decrease of that observed in ADR alone treated cells) while the ADR dose able to kill 50% of the $ADR^R$ cells exposed to both agents fell to 0.007 $\mu$M (about a 1,300-fold decrease). The selectivity elicited by LND on the ADR-resistant cell line appeared also by the significant decrease in the resistance index (0.875 for ADR + LND exposure *vs* 300 for ADR exposure).

Tables II and III show the intracellular ADR content in both tumour lines upon ADR and ADR + LND treatments. In order to compare cellular ADR content and drug activity, the corresponding cell surviving fractions were also reported. The results are expressed as the mean ± s.e. of three separate determinations. Remarkably, differences in ADR intracellular content were observed between the two MCF-7 cell lines.

In the sensitive cell line treated with ADR as a single agent, an increase of the ADR intracellular content as a function of both dose and exposure time was observed (Table II). The statistical analysis performed comparing the ADR content for each dose at the same exposure time demonstrated that there was a significant difference between each dose, with a $P$ value ranging from 0.0002 to 0.04. A statistical significance was also obtained comparing the ADR content for each exposure time at the same dose ($P$ value ranging from 0.0002 to 0.03).

Otherwise, in the resistant line treated with ADR alone the intracellular ADR content was not significantly modified by increasing both the dose and the exposure time (Table III). In fact, the intracellular ADR contents analysed either in function of doses or times did not significantly differ ($P$ values = 0.05–0.55). Significant differences were only observed comparing the intracellular ADR content at the lowest and highest ADR doses ($0.0007 \leqslant P$ value $\leqslant 0.01$) and exposure times ($0.003 \leqslant P$ value $\leqslant 0.03$).

Correspondence: G. Zupi, Regina Elena Institute, V.le Regina Elena 291, 00161 Rome, Italy.
Received 16 January 1991; and in revised form 1 May 1991.

**Table I** Enhancement of ADR-induced cytotoxicity by Lonidamine in MCF-7 WT and MCF-7 ADR$^R$ cells

| Drug | $IC_{50}$ (μM) MCF-7 WT | MCF-7 ADR$^R$ | Resistance index |
|---|---|---|---|
| ADR | 0.030 ± 0.012 | 9.000 ± 0.500 | 300 |
| ADR + LND | 0.008 ± 0.001 | 0.007 ± 0.001 | 0.875 |

Cytotoxicity of ADR in the presence and absence of LND, at a non-cytotoxic dose, was measured by determining the mean $IC_{50}$ values ± s.e. obtained from at least two separate experiments, each done in triplicate. Resistance index was calculated by dividing the $IC_{50}$ values of the resistant line by those of the parental line.

Figure 1 shows the intracellular ADR content of the two cell lines treated with the highest ADR dose as a function of exposure time. The trend of the two curves illustrates the different behaviour showed by the sensitive and the resistant lines. ADR concentration in WT cells increased steadily reaching the value of 78 ng ADR $10^{-6}$ cells within 72 h. On the contrary, in ADR$^R$ cells no marked increase in the drug content was observed during the treatment. These results demonstrate that the accumulation rate is consistently reduced in ADR$^R$ cells, indicating that the increase of drug doses and exposure times could be uneffective on drug-resistant tumour cells.

The ADR + LND combination differentially influenced the intracellular ADR accumulation of the two cell lines employed. Sensitive cells treated with the combination ($A_1$, $B_1$, $C_1$, $D_1$; Table II) showed values of drug concentration similar to those obtained after their exposure to ADR as a single agent (A, B, C, D; Table II). In fact as also demonstrated by the statistical analysis, the differences in ADR content between the two treatments were not statistically significant. These data correlate with the cell response to the combination; ADR + LND gave rise to a moderate enhancement of ADR cytotoxicity, as shown by the values of cell survival reported in Table II.

On the contrary, the ADR + LND association strongly affected the intracellular ADR accumulation of the resistant cells. In fact, the presence of LND nearly allowed to double the intracellular ADR content for all doses and exposure times employed. The differences of intracellular ADR level detected between the two treatments came out highly significant from the statistical analysis (A, B, C, D *vs* $A_1$, $B_1$, $C_1$, $D_1$; Table III).

Comparing the data reported in Tables II and III, it appears that the combination allowed ADR to achieve in the resistant cells an intracellular amount similar to that obtained for the wild type cells, indicating that LND had the ability to restore the *in vitro* sensitivity of ADR-resistant cells. In particular, LND allows ADR to reach the same intracellular content otherwise achievable with a 1,000-fold higher ADR dose given as single agent (Table III). The values of cell survival demonstrate the ability of LND to overcome ADR resistance (Table III). This effect is particularly relevant since at the lowest ADR dose, uneffective on the resistant cells, the combination determines a remarkable increase in cell lethality. In conclusion, these results indicate that the combination ADR + LND could be useful to improve the therapeutic index of ADR treatment.

The enhancement of the ADR cytotoxicity observed on the MCF-7 ADR$^R$ cells could be the result of two simultaneous selective LND effects: (1) the LND-lipid bilayer interactions give rise to clustering of intrinsic intramembrane proteins like P-170 glycoprotein, which is present on the MCF-7 ADR$^R$ cell membranes (Fairchild *et al.*, 1987), impairing its biological functions and thus reducing drug efflux and detoxification; (2) Lonidamine specifically inhibits the activity of mitochondria bound hexokinase (Floridi *et al.*, 1989) affecting the aerobic glycolysis of tumour cells. Since ADR$^R$ cells show an enhanced rate of glycolysis (3-fold), as compared to drug sensitive cells (Kaplan *et al.*, 1990), LND could selectively impair cellular energy-dependent mechanisms like drug efflux, drug-conjugation, enzyme synthesis, and lethal damage recovery.

**Table II** Intracellular ADR content and surviving fractions of MCF-7 WT cells exposed to ADR or to the association ADR + LND

| Treatment | Intracellular ADR content$^a$ (ng ADR $10^{-6}$ cells) 1 h | 12 h | 24 h | 72 h | Surviving cells$^b$ (% of control) |
|---|---|---|---|---|---|
| (A) ADR 0.01 μM | 24.0 ± 3.4 | 27.4 ± 3.0 | 30.0 ± 3.0 | 45.3 ± 3.2 | 46 ± 2.0 |
| ($A_1$) ADR 0.01 μM + LND$^c$ | 27.0 ± 1.6 | 30.0 ± 2.2 | 34.0 ± 1.5 | 46.0 ± 3.0 | 24 ± 1.5 |
| (B) ADR 0.1 μM | 27.5 ± 2.7 | 29.4 ± 1.4 | 32.2 ± 3.4 | 50.0 ± 2.3 | 30 ± 2.2 |
| ($B_1$) ADR 0.1 μM + LND | 28.0 ± 2.9 | 35.0 ± 1.8 | 36.0 ± 3.2 | 56.0 ± 2.5 | 21 ± 1.5 |
| (C) ADR 1 μM | 33.0 ± 2.6 | 42.0 ± 4.0 | 45.5 ± 3.2 | 67.0 ± 4.0 | 23 ± 1.6 |
| ($C_1$) ADR 1 μM + LND | 35.0 ± 3.1 | 42.0 ± 2.6 | 44.0 ± 3.4 | 68.0 ± 3.4 | 15 ± 1.7 |
| (D) ADR 10 μM | 42.5 ± 3.5 | 49.0 ± 4.9 | 59.0 ± 3.5 | 78.0 ± 3.5 | 18 ± 1.2 |
| ($D_1$) ADR 10 μM + LND | 40.0 ± 2.6 | 44.5 ± 3.0 | 57.0 ± 4.0 | 72.0 ± 2.3 | 12 ± 1.5 |

$^a$ADR content was evaluated at different doses and after different exposure times (1, 12, 24, 72 h). $^b$Surviving fractions were determined at the end of the experiment. $^c$LND = 50 μg $ml^{-1}$ percent survival of LND treated cells was 95 ± 0.6. Not statistically different: A *vs* $A_1$ $P \leqslant 0.8$; B *vs* $B_1$ $P \leqslant 0.9$; C *vs* $C_1$ $P \leqslant 0.8$; D *vs* $D_1$ $P \leqslant 0.5$.

**Table III** Intracellular ADR content and surviving fractions of MCF-7 ADR$^R$ cells exposed to ADR or to the association ADR + LND

| Treatment | Intracellular ADR content$^a$ (ng ADR $10^{-6}$ cells) 1 h | 12 h | 24 h | 72 h | Surviving cells$^b$ (% of control) |
|---|---|---|---|---|---|
| (A) ADR 0.01 μM | 12.0 ± 1.8 | 14.0 ± 2.5 | 18.0 ± 1.4 | 22.0 ± 2.7 | 100 ± 1.1 |
| ($A_1$) ADR 0.01 μM + LND$^c$ | 21.0 ± 1.7 | 25.0 ± 2.0 | 28.5 ± 2.0 | 33.0 ± 2.0 | 12 ± 1.9 |
| (B) ADR 0.01 μM | 15.0 ± 2.0 | 20.5 ± 1.6 | 22.0 ± 1.5 | 25.0 ± 2.9 | 94 ± 0.8 |
| ($B_1$) ADR 0.1 μM + LND | 29.0 ± 2.2 | 33.0 ± 2.6 | 35.0 ± 2.4 | 38.0 ± 1.5 | 11 ± 1.5 |
| (C) ADR 1 μM | 18.0 ± 1.4 | 22.5 ± 1.4 | 25.0 ± 1.2 | 28.0 ± 2.3 | 92 ± 3.0 |
| ($C_1$) ADR 1 μM + LND | 33.0 ± 1.4 | 35.0 ± 1.6 | 38.0 ± 1.0 | 39.5 ± 1.9 | 7 ± 1.1 |
| (D) ADR 10 μM | 25.0 ± 2.1 | 27.0 ± 1.3 | 29.5 ± 1.6 | 30.0 ± 1.8 | 48 ± 1.0 |
| ($D_1$) ADR 10 μM + LND | 37.0 ± 2.0 | 39.0 ± 1.7 | 42.0 ± 2.0 | 45.0 ± 1.9 | 6 ± 0.6 |

$^a$ADR content was evaluated at different doses and after different exposure times (1, 12, 24, 72 h). $^b$Surviving fractions were determined at the end of the experiment. $^c$LND = 50 μg $ml^{-1}$ percent survival of LND treated cells was 94 ± 0.4. Statistically different: A *vs* $A_1$ $P \leqslant 0.005$; B *vs* $B_1$ $P \leqslant 0.006$; C *vs* $C_1$ $P \leqslant 0.002$; D *vs* $D_1$ $P \leqslant 0.002$.

**Figure 1** Uptake of 10 μM ADR in (●) MCF-7 WT and (▲) MCF-7 ADR[R] cells. Drug uptake was evaluated at different periods of time. Each point is the mean ± s.e of three separate determinations.

The relationship between the high rate of aerobic glycolysis and drug resistance of MCF-7 ADR[R] cells was recently demonstrated by Kaplan *et al.* using 2-deoxyglucose, a specific glycolysis inhibitor. This drug was found to be extremely toxic for the cells which had acquired resistance to Adriamycin (Kaplan *et al.*, 1990). These data are in agreement with our results, demonstrating that a specific inhibitor of aerobic glycolysis like Lonidamine can play a remarkable role to reduce or overcome multidrug resistance.

In conclusion, the present study indicates the possibility to use Lonidamine as potentiating agent to interfere with MDR properties. Considering its good tolerance reported in preliminary clinical trials (Ozols *et al.*, 1983; Evans *et al.*, 1984; Pronzato *et al.*, 1989) Lonidamine, in combination with antitumour drugs, could improve cancer therapy when tumours initially responsive to chemotherapy develop resistance to the drugs following treatment with one of them.

This work was partially supported by grants from 'Ministero della Sanita' and AIRC (Italian Association for Cancer Research).

### References

BRADLEY, G., JURANKA, P.F. & LING, V. (1988). Mechanism of multidrug resistance. *Biochem. Biophys. Acta.*, **948**, 87.

CITRO, G., VERDINA, A., GALATI, R. & FLORIDI, A. (1988). Quantitation of adriamycin content by a sensitive immunochemical assay. *Anticancer Res.*, **8**, 549.

CURT, G.A., CLENDENNIN, N.J. & CHABNER, B.A. (1984). Drug resistance in cancer. *Cancer Treat. Rep.*, **68**, 87.

EVANS, W.K., SHEPPARD, F.A. & MULLIS, B. (1984). Phase II evaluation of Lonidamine in patients with advanced malignancies. *Oncology*, **41**, 69.

FAIRCHILD, C.R., IVY, S.P., SAO-SHAN, C.S. & 6 others (1987). Isolation of amplified and overexpressed DNA sequences from Adriamycin-resistant human breast cancer cells. *Cancer Res.*, **47**, 5141.

FLORIDI, A. & LEHNINGER, A. (1983). Action of the antitumor and antispermatogenic agent Lonidamine on electron transport in Ehrlich ascites tumor mitochondria. *Arc. Biochem. Bioph.*, **226**, 73.

FLORIDI, A., PAGGI, M.G. & FANCIULLI, M. (1989). Modulation of glycolysis in neuroepithelial tumors. *J. Neurosurg. Sci.*, **33**, 55.

JULIANO, R.L. & LING, V. (1976). A surface glycoprotein modulating drug permeability in Chinese hamster ovary cells mutants. *Biochem. Biophys. Acta.*, **455**, 152.

KAPLAN, O., NAVON, G., LYON, R.C., FAUSTINO, P.J., STRAKA, E.J. & COHEN, J.S. (1990). Effects of 2-deoxyglucose on drug-sensitive and drug-resistant human breast cancer cells: toxicity and magnetic resonance spectroscopy studies of metabolism. *Cancer Res.*, **50**, 544.

KIM, J.H., ALFIERI, A., KIM, S.H., YOUNG, C.W. & SILVESTRINI, B. (1984). Radiosensitization of Meth-A Fibrosarcoma in mice by Lonidamine. *Oncology*, **41**, 36.

LYON, R.C., COHEN, J.S., FAUSTINO, P.J., MEGNIN, F. & MYERS, C. (1988). Glucose metabolism in drug-sensitive and drug-resistant human breast cancer cells monitored by magnetic resonance spectroscopy. *Cancer Res.*, **48**, 870.

MALORNI, W., ARANCIA, G., DE MARTINO, C. & 4 others (1988). On the mechanism of action of Lonidamine: a study on human erythrocytes membrane. *Exp. Mol. Pathol.*, **49**, 361.

OZOLS, R.F., DEISSEROTH, A.B., JAVADPOUR, N., BARLOCK, A., MESSERSCHMIDT, J. & YOUNG, R.C. (1983). Treatment of poor prognosis nonseminomatous testicular cancer with high dose platinum combination chemotherapy regimen. *Cancer*, **51**, 1803.

PRONZATO, P., AMOROSO, D., BERTELLI, G. & 6 others (1989). Phase II study of Lonidamine in metastatic breast cancer. *Br. J. Cancer*, **59**, 251.

RAAPHORST, G.P., FEELEY, M.M., HELLER, D.P. & 4 others (1990). Lonidamine can enhance the cytotoxic effect of cisplatin in human tumor cells and rodent cells. *Anticancer Res.*, **10**, 923

VAN DER BLIEK, A.M. & BORST, P. (1989). Multidrug resistance. *Adv. Cancer Res.*, **52**, 165.

ZUPI, G., GRECO, LAUDONIO, N., BENASSI, M., SILVESTRINI, B. & CAPUTO, A. (1986). *In vitro* and *in vivo* potentiation by Lonidamine of the antitumor effect of Adriamycin. *Anticancer Res.*, **6**, 1245.